# The Cancer Genome Atlas Clinical Explorer: a web and mobile interface for identifying clinical–genomic driver associations

**DOI:** 10.1186/s13073-015-0226-3

**Published:** 2015-10-27

**Authors:** HoJoon Lee, Jennifer Palm, Susan M. Grimes, Hanlee P. Ji

**Affiliations:** Division of Oncology, Department of Medicine, Stanford University School of Medicine, Stanford, CA 94305 USA; Stanford Genome Technology Center, Stanford University, Palo Alto, CA 94304 USA

## Abstract

**Background:**

The Cancer Genome Atlas (TCGA) project has generated genomic data sets covering over 20 malignancies. These data provide valuable insights into the underlying genetic and genomic basis of cancer. However, exploring the relationship among TCGA genomic results and clinical phenotype remains a challenge, particularly for individuals lacking formal bioinformatics training. Overcoming this hurdle is an important step toward the wider clinical translation of cancer genomic/proteomic data and implementation of precision cancer medicine. Several websites such as the cBio portal or University of California Santa Cruz genome browser make TCGA data accessible but lack interactive features for querying clinically relevant phenotypic associations with cancer drivers. To enable exploration of the clinical–genomic driver associations from TCGA data, we developed the Cancer Genome Atlas Clinical Explorer.

**Description:**

The Cancer Genome Atlas Clinical Explorer interface provides a straightforward platform to query TCGA data using one of the following methods: (1) searching for clinically relevant genes, micro RNAs, and proteins by name, cancer types, or clinical parameters; (2) searching for genomic/proteomic profile changes by clinical parameters in a cancer type; or (3) testing two-hit hypotheses. SQL queries run in the background and results are displayed on our portal in an easy-to-navigate interface according to user’s input. To derive these associations, we relied on elastic-net estimates of optimal multiple linear regularized regression and clinical parameters in the space of multiple genomic/proteomic features provided by TCGA data. Moreover, we identified and ranked gene/micro RNA/protein predictors of each clinical parameter for each cancer. The robustness of the results was estimated by bootstrapping. Overall, we identify associations of potential clinical relevance among genes/micro RNAs/proteins using our statistical analysis from 25 cancer types and 18 clinical parameters that include clinical stage or smoking history.

**Conclusion:**

The Cancer Genome Atlas Clinical Explorer enables the cancer research community and others to explore clinically relevant associations inferred from TCGA data. With its accessible web and mobile interface, users can examine queries and test hypothesis regarding genomic/proteomic alterations across a broad spectrum of malignancies.

**Electronic supplementary material:**

The online version of this article (doi:10.1186/s13073-015-0226-3) contains supplementary material, which is available to authorized users.

## Background

Extensive catalogues of genetic aberrations in cancers have been generated by high throughput technologies such as next-generation sequencing (NGS) and genomic scale microarrays [1–3]. For example, over 800 genomes [4] and 2,700 exomes [5] from more than 25 cancer types have been sequenced by NGS since 2008 [6]. Despite the breadth and depth of these cancer genome data sets, there are only a small number of studies that utilize these cancer genome data sets for identifying associations among genomic findings and clinical parameters or phenotypes. Rather, the majority of studies use unsupervised analysis methods to delineate specific molecular signatures [7–11]. Many of these studies have restricted sample sizes, thus the studies have limited power in detecting genomic associations with various clinical phenotypes [12, 13]. Although molecular profiling studies have brought enormous biological insights about cancer, clinical translation of these discoveries requires associating molecular features with clinical phenotypes.

The Cancer Genome Atlas (TCGA) project has generated genomic, epigenomic, transcriptomic, and proteomic data for over 20 different cancer types [14–21]. These data sets provide broad insight into the underlying genetic aberrations existing across multiple cancer types. In addition, TCGA has clinical data describing specific metrics such as histopathology and clinical stage, among others. Overall, TCGA data has the potential for determining the clinical significance of critical genetic aberrations.

For clinicians and other cancer researchers lacking bioinformatics expertise, extrapolating desired information from the copious amounts of data supplied by TCGA proves to be a difficult task. Several websites, including the cBio portal [22] and the University of California, Santa Cruz (UCSC) genome browser [23], were developed to make TCGA data more accessible. These sites are generally configured for providing primary genomic results rather than clinical associations. Some programs, such as StratomeX, use an unsupervised approach to explore the relationship between clinical parameters and patient stratifications based on molecular profiling [24]. However, the results from StratomeX are provided as tumor sample clusters without the granularity of identifying specific genes. In contrast, many investigators are interested in reviewing lists of candidate genes that facilitates the interpretation of genomic results for non-computational biomedical researchers and other users.

To enable a gene-centric exploration of the potential clinical–genomic associations in TCGA data, we developed the Cancer Genome Atlas Clinical Explorer (http://genomeportal.stanford.edu/pan-tcga/). Enabling improved access of cancer genomic data, this web and mobile interface allows users to navigate the list of cancer genes, micro RNAs (miRs), or proteins from TCGA data and explore their translational or clinical significance. We conducted a successful initial study [25] where we analyzed the relationship between genomic/proteomic profiles and clinical phenotypes for colorectal cancers using the breadth of TGCA data. Using an elastic-net regularized regression method we integrated genomic alteration data from different genomic platforms as well as clinical meta-data from TCGA. For example, for colorectal cancer, the elastic-net analysis identified hyper-methylation of *MLH1* and mutations of *TGFBR2* as top predictors for a tumor with microsatellite instability (MSI)—these are well-known examples of MSI-related events. Subsequently, we identified genetic aberrations in cancer genes indicative of clinical stage in colorectal cancer, considering multiple genomic features and clinical data. We determined that combining data from multiple genomic platforms outperformed the analysis based on an individual genomic assay.

Given our success in the small pilot study, we conducted a new and significantly expanded study using 25 cancer types with 18 clinical parameters from TCGA Project. Our results from these elastic-net analyses successfully identified known associations between genomic/proteomic and clinical data.

The Cancer Genome Atlas Clinical Explorer allows users to answer queries such as “which genes correlate with the metastasis of skin cancer,” “do stomach cancers with *PIK3CA* genetic aberrations behave differently in EBV [Epstein–Barr virus] infected individuals compared to uninfected,” or “what are the differences in *TP53* copy number between tumor samples with or without *TP53* mutations.” Overall, this web interface eliminates barriers to accessing TCGA data, allows researchers to address important questions to their projects, and allows researchers to adjust their hypotheses and experimental designs in the investigations accordingly.

## Construction and content

All data originated from the public websites of TCGA Project. The Cancer Genome Atlas Clinical Explorer summarizes TCGA clinical parameters and translates these data into a list of clinically relevant cancer drivers including genes, miRs, and proteins. First, we generated descriptive statistics such as mutation frequencies or copy number variation (CNV). These selected gene aberration statistics were categorized by cancer types and derived from SQL queries using our relational database that contains pre-processed TCGA data, as described later. Second, we generated a list of genes, miRs, and proteins that correlate with specific clinical parameters using elastic-net analysis as described [25]. For example, if breast cancer data had ten clinical parameters with an adequate number of samples having annotation, the elastic-net analysis would be run separately for each clinical parameter. Overall, our analysis included 25 cancer types and 18 clinical parameters.

Some of the clinical features were available to a limited number of cancer types. For instance, PAM50 information is only available in breast cancer samples and EBV infection is exclusive to stomach cancer. Compared to our initial, limited analysis on TCGA colorectal cancer data, this new study has been dramatically increased in scale and fully leverages the wealth of new molecular data, clinical parameters, and different cancer types. For example, new features of this study include (1) an expanded miR and reverse phase protein array (RPPA) data set that was not previously available; (2) analysis of an additional 24 cancers with more than ten clinical parameters, providing a significantly more expanded analysis and results database compared to our previous publication (e.g. four clinical parameters in colorectal adenocarcinoma [COADREAD]); and (3) development of a new interactive interface that allows users to easily explore TCGA data with an orientation toward clinical phenotypes.

### Data sources

We downloaded TCGA genomic/proteomic data (2 April 2015 version) from the Broad Firehose (http://gdac.broadinstitute.org) using firehose_get (version 0.4.3) and ran md5sum to ensure the integrity of the downloaded data and to verify that all genomic data files were intact. These data files included genomic, transcriptomic, epigenomic, and proteomic data for each of the 25 cancer types. Specifically, these data included DNA CNV, somatic mutations, mRNA expression level by RNA sequencing (RNA-Seq), DNA methylation, miR expression level by RNA-Seq, and protein expression level by RPPA (Table 1).

**Table 1:** Sample numbers of clinical data used from The Cancer Genome Analysis pan-cancer data set

Tumor type (TCGA ID)		Number of samples in analysis			Number of clinical parameters
	Total	Gene	micro RNA	Protein	
Adrenocortical carcinoma (ACC)	92	75	80	46	5
Urothelial bladder cancer (BLCA)	412	388	409	127	8
Breast invasive carcinoma (BRCA)	1098	945	755	410	9
Cervical cancer (CESC)	307	190	307	173	7
Colorectal adenocarcinoma (COADREAD)	628	476	295	461	10
Esophageal cancer (ESCA)	185	0	184	126	9
Glioblastoma multiforme (GBM)	610	111	0	214	2
Head and neck squamous cell carcinoma (HNSC)	528	496	486	212	9
Chromophobe renal cell carcinoma (KICH)	66	66	66	0	4
Kidney renal clear cell carcinoma (KIRC)	537	446	254	454	7
Papillary kidney carcinoma (KIRP)	291	161	291	207	7
Acute myeloid leukemia (LAML)	200	160	0	0	1
Lower grade glioma (LGG)	516	510	512	258	4
Liver hepatocellular carcinoma (LIHC)	377	189	372	0	7
Lung adenocarcinoma (LUAD)	582	485	450	181	9
Lung squamous cell carcinoma (LUSC)	504	178	342	195	8
Ovarian serous cystadenocarcinoma (OV)	605	216	453	412	1
Pancreatic ductal adenocarcinoma (PAAD)	185	145	178	106	9
Pheochromocytoma and paraganglioma (PCPG)	179	161	179	79	1
Prostate adenocarcinoma (PRAD)	498	419	494	0	4
Skin cutaneous melanoma (SKCM)	470	290	351	169	6
Stomach adenocarcinoma (STAD)	443	255	395	264	11
Thyroid carcinoma (THCA)	503	397	502	222	7
Uterine corpus endometrioid carcinoma (UCEC)	559	241	411	200	4
Uterine carcinosarcoma (UCS)	57	56	56	48	1
Total cancer = 25	10,432	7,056	7,822	4,564	

*TCGA* The Cancer Genome Atlas

Clinical and pathological data covering 18 clinical parameters were obtained from TCGA. During the course of the study, we noted that the availability and comprehensiveness of clinical data varied across the cancer types. For example, the status of EBV infection was only reported for stomach cancer and clinical stage was only listed for 16 of the 25 cancers in the TCGA data set we analyzed. Given the fragmented nature of these clinical metric data sets, we consolidated the different clinical metrics across several sources. Twelve clinical parameters were obtained from the public TCGA data portal, five clinical parameters were acquired from the UCSC cancer genome browser, and one clinical parameter was obtained from the cBio Portal (Table 2). Data consistency was then evaluated across these sources. When inconsistencies or issues among the sources were identified, adjustments and resolutions were made. For example, although TCGA data portal provides multiple files for each patient, there were 71 cases where the values for a single patient were not consistent (Additional file 1: Table S1). These cases are annotated with “NA” as a missing value. In another example, we only annotated breast cancer samples regarding triple markers (her2, estrogen, and progesterone) when this information was available. Subsequently, we classified these breast cancer samples into four molecular subtypes: triple positive, Her2 positive, ER positive (either estrogen or progesterone positive, or both), and triple negative.

**Table 2:** Type, subtypes, and sources of clinical parameters used in elastic-net analysis. Eighteen total clinical parameters were included—availability of each clinical attribute is dependent on cancer type

Clinical parameter	Number of subtypes	Type	Subtypes	Number of cancer types	Source
Country	16	Categorical	US, Russia, Korea South, Italy, etc*.*	17	TCGA
Gender	2	Binary	male, female	18	TCGA
HistoType	by cancer type	Categorical	Ex) Intestinal/diffuse for stomach	10	TCGA
PriorMalignancy	2	Binary	yes, no	7	TCGA
FamilyHistory	by cancer type	Ordinal	0,1,2,3	1	TCGA
M-Status	2	Binary	M0, M1	15	TCGA
N-Status	4	Ordinal	N0, N1, N2, N3	18	TCGA
ClinicalStage	4	Ordinal	I, II, III, IV	16	TCGA
T-Status	5	Ordinal	T0, T1, T2, T3, T4,	18	TCGA
HistoGrade	3	Ordinal	Low, Intermediate, High	11	TCGA
SmokingHistory	4	Categorical	Current smoker, Lifelong Non-smoker, Current reformed smoker for >15 years, Current reformed smoker for ≤15 years	8	TCGA
MolecularSubtype	2	Categorical	CIN, GS, MSI, EBV	1	TCGA
MSIstatus	3	Ordinal	MSS, MSI-L, MSI-H	5	UCSC
PAM50clust	5	Categorical	Normal-like, Luminal A, Luminal B, Basal-like, HER2-enriched	1	UCSC
RPPAclustersBRCA	6	Categorical	ReacI, LumA/B, Basal, LumA, Her2, ReacII	1	UCSC
GeneExpSubtype	4	Categorical	Classical, Mesenchymal, Proneural, Neural	1	UCSC
TripleMarker	4	Categorical	TripleNegative, Her2Positive, Erpositive, TriplePositive	1	UCSC
EBV present	2	Binary	Positive, Negative	1	cBio

*cBio* cBio portal, *EBV* Epstein–Barr virus, *GeneExpSubtype* types based on gene expression in glioblastoma multiforme, *HistoGrade* histology grade, *HistoType* histological type, *CIN* chromosomal instability, *GS* genomically stable, *MSIstatus* microsatellite instability status, *MSS* Microsatellite stable, *MSI-L* Microsatellite instable-low, *MSI-H* Microsatellite instable-high, *PAM50clust* clusters based on PAM50, *RPPAclustersBRCA* clusters based on reverse phase protein array data, *TCGA* The Cancer Genome Atlas, *UCSC* University of California Santa Cruz cancer genome browser

Next, we categorized each clinical parameter into one of three types: categorical, ordinal, or binary. Categorical variables depict clinical parameters with multiple subtypes but no clear ordering (e.g., smoking history), ordinal describes clinical parameters with multiple subtypes with identifiable ordering (e.g., clinical stage), and binary represents clinical parameters with only two subtypes (e.g., gender). Finally, we produced a comprehensive data table for all 18 clinical parameters across all of 25 cancer types. These lists can be reviewed and downloaded at our web portal (http://genomeportal.stanford.edu/pan-tcga/data_download).

### Target selection for elastic-net analysis

To increase the signal of driver events versus non-informative passengers, we vetted the gene list for the elastic-net analysis. We included known and putative cancer genes according to the Catalogue of Somatic Mutations in Cancer (COSMIC) [1] and results from various TCGA studies. As of February 2015, the COSMIC database listed 547 genes as cancer-related owing to their implication for a role in cancer biology as documented by the scientific literature. We also included 135 genes currently targeted by drugs according to the database tumor alterations relevant for genomics-driven therapy (TARGET; www.broadinstitute.org/cancer/cga/target) (Additional file 2: Table S2). In addition, we included genes with significant mutations (MutSig; 852), focal amplifications (CN-AmpPeak; 502), and focal deletions (CN-DelPeak; 2,105) that were reported by Broad Firehose from TCGA data for all 25 cancers (Additional file 3: Table S3). A total of 2,180 cancer genes from both COSMIC and TCGA were selected for analysis (Additional file 4: Table S4). For the miR-oriented and protein-oriented supervised analysis, we included all 1,751 miRs that were presented in miRNA-Seq data and all 228 proteins that were presented in RPPA data from the 25 cancers types we selected to analyze (Additional file 4: Table S4). We included all miRs and proteins because of the limited list that is currently available for these platforms; TCGA pre-selected these candidates. For example, the RPPA assay technology is constrained by the number of different proteins that can be measured.

### Data pre-processing and normalization

**Fig. 1:**
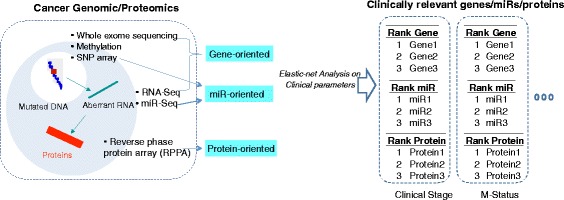
Overview of the elastic-net analysis pipeline. Genomic data was downloaded from Broad Firehose and analyzed in three separate groups. Gene-oriented analysis relied on samples with data for mutations, copy number alterations, RNA-Seq, and methylation. The genes, miRs, and proteins with >3 % missing values were excluded; otherwise missing values were imputed using the median sample value. MicroRNA (*miR*) and proteins (reverse phase protein array, *RPPA*) were analyzed separately given the smaller number of genes and targets that came from these analysis platforms. Integrated genomic/proteomic matrices were associated with clinical outcomes by elastic-net across all 25 type of cancer. *SNP* single nucleotide polymorphism

We formatted raw genomic/proteomic TCGA data to the updated, filtered, normalized, and structured meta-data by each platform (Fig. 1). First, we updated every genomic symbol to HUGO Gene Nomenclature (HGNC, June 2015 version) and revised all protein names to match those assigned from the primary output of the Broad Firehose. Fifteen gene symbols were removed, because they did not have current HUGO identifiers (Additional file 5: Table S5).

Second, we selected those samples that underwent analysis using all of the available genomic platforms. This included gene-oriented analysis (CNV, mutations, RNA-Seq, methylation), miR analysis (CNV, RNA-Seq), and protein analysis (RPPA). Of note, in gene-oriented analysis, all of the samples had methylation values that were determined with two platforms, Infinium HumanMethylation27 (HM27) and/or Infinium HumanMethylation450 (HM450). To increase sample coverage, probes that were common to both platforms were placed into a methylation matrix—this approach was completed in eight cancers including BRCA, COADREAD, GBM, KIRC, LUAD, LUSC, STAD, and UCEC. LAML was the only exception. For this cancer, all the samples had been analyzed on both platforms and, for this reason, we exclusively used the HM450 methylation platform given that this version of the assay is more comprehensive than the HM27 methylation platform.

Third, we removed any molecular features measurements that were missed from 3 % or more samples and replaced missing values with the median across all samples for each feature. In average, 257 genes (for RNA-Seq) and 327 probes (for methylation), 621 miRs, and no protein were excluded from analysis, while imputation occurred with 448 genes in RNA-Seq, 289 probes in methylation, and 357 miRs from miR-Seq. Proteins were not excluded given the completeness of the data. The list of excluded gene features can be reviewed and downloaded at our web and mobile portal (http://genomeportal.stanford.edu/pan-tcga/data_download).

Fourth, as has been done with other studies, we normalized the scale of each feature by the standard deviation of each gene’s measurement plus the tenth percentile of the global standard deviation in each genomic/proteomic assay [25, 26], as follows:

$$ \widehat{\mathrm{g}}\left(\mathrm{i},\mathrm{j}\right)=\frac{\mathrm{g}\left(\mathrm{i},\mathrm{j}\right)}{\mathrm{sd}\left(\mathrm{g}\left(\mathrm{i}\right)\right)+\mathrm{s}{\mathrm{d}}_{10}\left(\mathrm{g}\right)} $$

where g(i,j) is the value for feature i in sample j, sd(g(i)) is the standard deviation across samples for feature i, sd_10_(g) is the tenth percentile value of standard deviations across features, and ĝ(i, j) is the normalized feature value. This standard deviation correction factor is standard in microarray analysis [26] and minimizes the risk of generating outliers due to normalization. The scale of each platform was also normalized.

To execute the regression analysis, we converted clinical outcome values into an integer according to the type of clinical parameter: ordinal, binary, or categorical (Table 2). For ordinal and binary, we converted clinical outcomes into numerical values (Additional file 6: Table S6). For example, Stage I, II, III, and IV designations were converted into integer values of 1, 2, 3, and 4 respectively. Citing another example, female or male sex annotations were altered to either 0 or 1. Categorical clinical features were converted into binary types by comparing one class to the remaining classes. For example, there are four molecular subtypes in breast cancer: triple positive, Her2 positive, ER positive, and triple negative. Thus, using these four designated subtypes, we complete the following multiple binary comparisons: triple negative subtype versus others, Her2 positive versus others, ER positive versus others, and triple positive versus others. We then converted a selected class into 1 and others into 0 to achieve an integer measurement. These converted clinical outcomes were assigned to the samples in the genomic/proteomic data matrices as a dependent variable for elastic-net analysis. Samples without available clinical metrics and outcomes were excluded from analysis.

### Identification of genes/miRs/proteins associated with clinical phenotype

As described previously, we organized the pre-processed data into three groups: (1) gene-oriented; (2) miR-oriented; and (3) protein-oriented (Fig. 1). We used elastic-net regression to estimate an optimal multiple linear regression of the clinical outcome on the space of genomic features from these three data groups. For example, because there were 11 available clinical parameters in stomach cancer, we conducted elastic-net analysis 33 times (three groups × 11 clinical parameters) for stomach cancer. Our analysis relied on all of the available clinical attributes across all 25 types of cancers.

We used the elastic-net algorithm package available in MATLAB (MathWorks, Natick, MA, USA) as previously published [25]. There were three distinct data categories, organized into separate data matrices. First, we compiled and integrated four genomic data types (DNA CNV, somatic mutations, mRNA expression level by RNA-Seq, and DNA methylation) for gene-oriented data. Second, we analyzed the miRNA-oriented data set using miRNA genomic CNV and miRNA expression level by RNA-Seq. Third, we used proteomic information available from the RPPA data.

We rescaled each feature and included the data into a single integrated matrix. Briefly, each feature in a matrix was normalized by both the standard deviation of each gene’s value and the tenth percentile of the global standard deviations. The elastic-net regression estimates an optimal multiple linear regression of the clinical outcome on the integrated space of genomic/proteomic features. For each supervised analysis, it calculates the coefficient values associated with each genomic feature while limiting the number of predictors in the model to ensure the selected model is general.

To confirm each supervised comparison, we used 10-fold cross validation to identify the set of genes/miRs/proteins that minimized the average mean-squared error on each testing set. The resulting coefficients from the regularized regression were used to rank genomic/proteomic features by their association with clinical attributes. The features were scored proportionally to their ranks and the score of each gene is the sum of all scores of its selected features. Nonparametric bootstrap resampling was used to assess the robustness of the set of top-ranked genes to changes in the training data as has been previously validated. The complete data set was resampled with replacement up to 2,000 times and the elastic-net regression was recomputed for each bootstrap data set. Features that are consistently selected by the bootstrap regression have high rank and low variance. Genes that are highly ranked for individual category of genetic aberration (e.g. mutations) or show high ranks among multiple different genomic assays are the most robust.

Lists of clinically relevant genes for the 25 cancer types were identified from elastic-net analysis. The number of candidate genes associated with clinical stage ranged from zero (ESCA) to 48 (THCA), with an overall average of 13.6 across the 16 cancer types. The number of miRs associated with clinical stage ranged from 0 (BRCA, ESCA, HNSC, KICH, LUAD, PAAD, STAD) to 46 (KIRP) with an average of 7.1. Finally, the number of proteins associated with clinical stage ranged from 0 (ACC, BRCA, LUAD, LUSC, STAD, KICH, LIHC) to 23 (KIRC) with an average of 3.4. A total of 199 gene-oriented, 111 miR-oriented, and 45 protein-oriented top candidates were found when analyzed with clinical stage. To directly query these candidates, the user types in the name of the genes/miRs/proteins of interest or by selecting pre-defined icons (see Utility and Discussion).

We provided statistical significance for genes, miRs, and proteins—among 10-fold cross validation of elastic-net analysis—for *P*-values <0.01. After identifying the candidate list from elastic-net analysis, we tested each candidate individually with the null hypothesis that there is no difference in a selected genomic feature between two groups by a clinical parameter with Bonferroni correction. Fisher’s exact test was used to assess significance for mutation and copy number data, while a Mann–Whitney–Wilcoxon Test was used to assess significance among RNA-Seq, methylation, miR-Seq, and RPPA data. As an example, our integrative elastic-net analysis identified 107 genes associated with clinical stage in STAD. We focused on the candidate gene *HEATR3* with the null hypothesis that there is no difference in copy number changes of *HEATR3* between early and advanced stage. We conducted a Fisher’s exact test using a 2 × 2 contingency table with four numbers: (1) number of samples with amplified *HEATR3* in stage I and II, (2) number of samples without amplified *HEATR3* in stage I and II, (3) number of samples with amplified *HEATR3* in stage III and IV, and (4) number of samples without amplified *HEATR3* in stage III and IV. To apply Bonferroni correction, we multiplied the *P-*value of *HEATR3* by 107, which was the number of tests for this specific analysis. The candidate genes were ones that had a corrected *P-*value less than 0.01. Among the 107 genes initially identified, only 24 had a corrected *P*-value less than 0.01. A link to download the list of full candidates selected by elastic-net analysis is still available (http://genomeportal.stanford.edu/pan-tcga/data_download).

This list may guide users to select targets for experiment validation. As an example, there are 24 genes associated with clinical stage in STAD. If users have a list of genes they are interested in, and seven of them are on our list, it is better to validate own genes of interest using our higher-ranked genes. Statistically speaking, a genetic alteration in a higher-ranked gene has a greater influence on clinical parameters than alterations in lower-ranked genes. Without any prior genes of interest, it may be better to validate experiments with the highest-ranked genes, such as top-ranked *HEART3*. The *P*-value is an indicator of how significantly these genomic features distinguish between limited and advanced stage cancer. If the user is interested in expression levels, *NTPX1* is the highest-ranked gene with regards mRNA expression.

### Database schema

All processed data mentioned above was migrated to a structured MySQL relational database from source-formatted files. The data were migrated using a combination of bash scripts and Rails rake tasks. The web application was written in Ruby on Rails, which is well suited for a relational backend database. We categorized the data according to the type or level of elastic-net analysis that was conducted. This included high-level clinical summaries, outcome summaries, and multiple other tables correlating samples, genes, proteins, miRs, and clinical parameters.

### Web implementation

The resulting data is queried, processed, and made viewable through a Ruby on Rails web application; Rails 4.0. Bootstrap is currently used for the front-end framework. The web application is hosted on Linux Ubuntu 10.04, Apache 2.2.14, with Passenger 4, Ruby 1.9.3. To provide a visual summary of data, Highcharts—a JavaScript charting library—was used to generate different types of charts and graphs on web pages. Each chart is dynamically generated (no charts are hard coded) using data returned from queries in the Rails controllers. These data are sorted, filtered, and processed, and in some cases statistical formulation is applied. The data are then passed on to the chart code by html5 data attributes to Highcharts. This enables the data to be rendered in page views. Some pages have multiple charts dynamically displayed, made possible with Ruby code in the Rails view templates.

## Utility and discussion

**Fig. 2:**
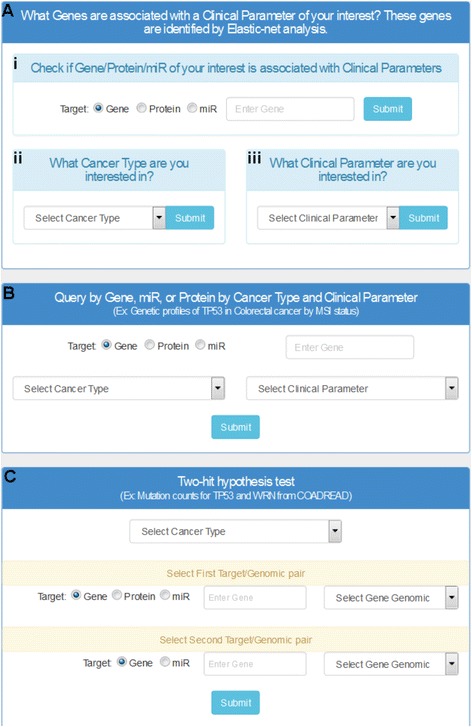
The Cancer Genome Atlas Clinical Explorer homepage. The web interface provides three different ways of navigating TCGA data. **a** Users can inquire about the clinical relevance of specific genes, miRs, or proteins identified by elastic-net analysis. This is done by entering the (*i*) gene name, (*ii*) cancer type, or (*iii*) clinical parameter. **b** Users can examine if a somatic alteration behaves differently between categories in a clinical parameter and in a cancer type. **c** Users can investigate how a genetic event affects another alteration in a selected cancer type using the two-hit hypotheses test

The Cancer Genome Atlas Clinical Explorer is a clinically oriented summary of genomic/proteomic data organized by cancer type or clinical parameters. Its interface enables users to query TCGA data in multiple ways (Fig. 2). First, users can search for clinically relevant gene/protein/miRs identified by elastic-net analysis. Second, users can query a gene, miR, or protein in subcategories of a selected clinical parameter in a chosen cancer of interest. Third, users can test a specific gene for results supporting the two-hit hypotheses.

As an indicator of the robustness of our results, we found that for the molecular subclass HER2-positive breast cancers, *ERRB2* and *HER2* were identified as top predictors from gene-oriented and protein-oriented analysis respectively. As an additional test regarding the overlapping correlations, we compared our study to a previous TCGA study focused on GBM [27]. We used 110 GBM samples from the TCGA for elastic-net analysis regarding GBM subtype. The TCGA study had more samples but limited clinical annotation, thus restricting the number of samples from which we could conduct our supervised analysis based on clinical parameters. When we used our elastic-net analysis using only one class of genomic aberration (e.g. mutation alone, copy number alone), our results were highly concordant with the results of the TCGA study in terms of molecular subclass.

We used only one genomic feature to facilitate a direct comparison with the TCGA results. When we used only the CNV data, our supervised analysis of the proneural molecular subclass compared to all others identified *OR51E2* and *OR52E4* (chr 11p15) as the second and third ranked candidates; *CDK4* was the 17th highest ranking CNV. This result is concordant with the TCGA study results regarding this molecular subclass. When we used only the mutation data, our supervised analysis of mesenchymal subclass identified *IDH1* and *TP53* as the first and second ranked candidates. Again, this result overlaps with the TCGA results. When we used copy number data, our supervised analysis of the classical subclass revealed *EGFR* as the top ranking candidate, a result that is concordant with the TCGA study. For the mesenchymal subclass, our results were concordant with TCGA in that we identified *NF1*, *CDH18*, and *RB1* as the top, tenth, and 18th candidates, primarily using mutation data, and *NF1* was also seen prominently in terms of somatic CNV. As the clinical annotation is extended to more GBM samples, we anticipate that our approach will identify more of the genes found in the original study and place them in the context regarding their association with clinical parameters.

### Search for clinically relevant genes/miRs/proteins

**Fig. 3:**
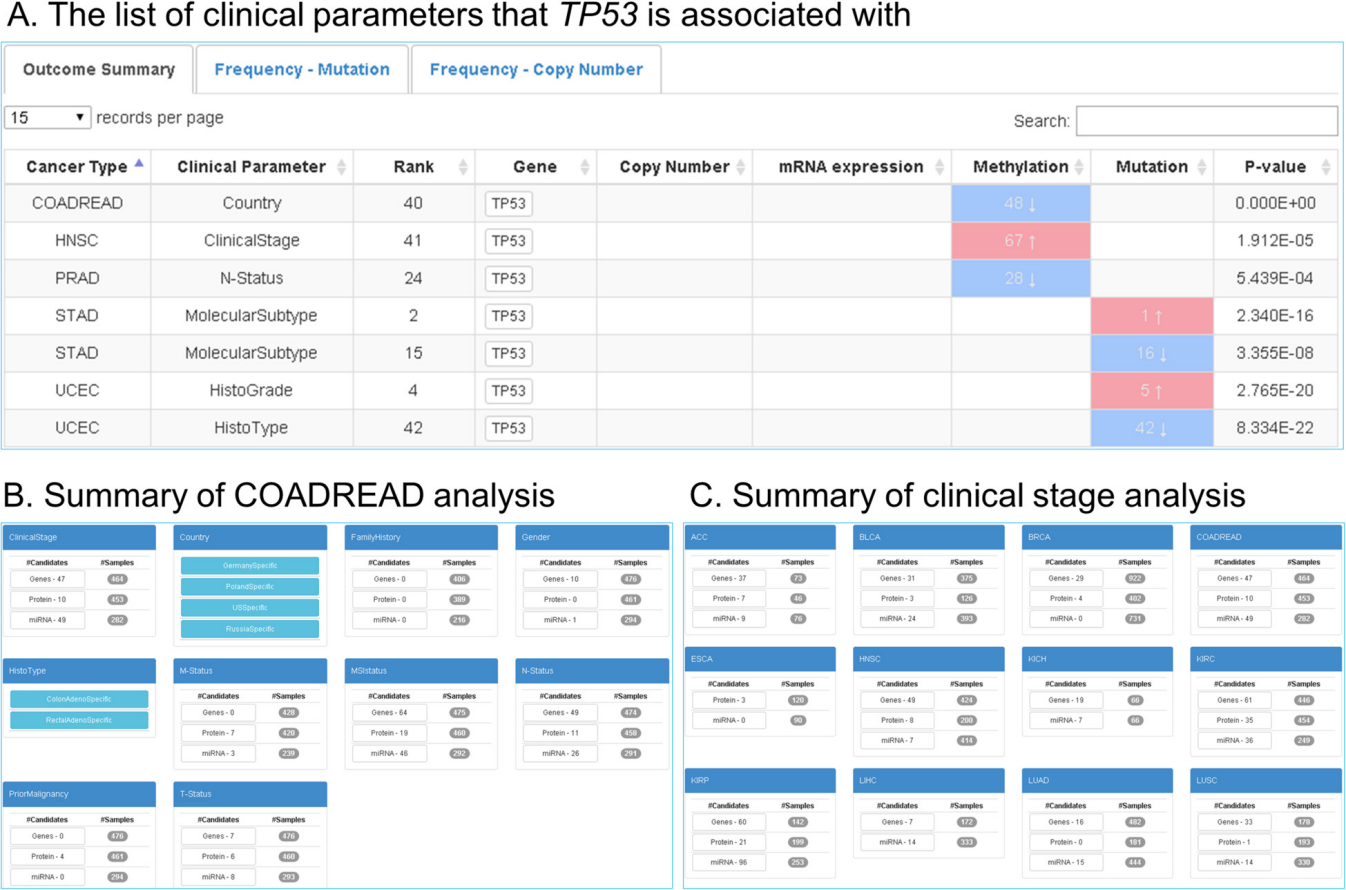
Query results page – clinically relevant genes, miRs, or proteins. **a** The search results page when *TP53* is entered in the search panel (Fig. 2a *i*) and the explorer website has retrieved data using elastic-net analysis. **b** The search results page for a specific cancer type; COADREAD is selected from the drop-down menu (Fig. 2a *ii*) and this action retrieves results about COADREAD. This includes summary tables for genes, miRs, and proteins potentially associated with ten clinical parameters in COADREAD. Each clinical parameter table displays the number of candidates (gene, miRs, and proteins) and the number of samples used in each analysis. Categorical clinical parameters list subtypes beneath the clinical parameter title; each subtype, when selected, displays a more complete summary table including number of candidates (gene, miRs, and proteins) and the number of samples used in each analysis. **c** The search results page when clinical stage is selected from drop-down menu (Fig. 2a *iii*). Results are displayed for summary tables across all of the cancers. Each clinical stage table displays the number of candidates (gene, miRs, and proteins) and the number of samples used in each analysis

As noted previously, the first search capability allows users to search by the genes/miRs/protein name (Fig. 2a*i*). Once a gene, protein, or miR is entered into the search window, a new page will display clinical parameters associated with their molecule of interest. For example, entering *TP53* will display the clinical parameters associated with *TP53* across all cancer types as identified by elastic-net analysis (Fig. 3a). In this search, users can also see the frequency of mutations and copy number changes on *TP53* across all cancer types located in separate tabs labeled “Frequency-Mutation” and “Frequency-Copy Number.” Sort functions for each column allows users to visualize that *TP53* is most frequently mutated, amplified, or deleted in OV, HNSC, and KICH respectively. A *P*-value is provided as well to enable users to sort based on statistical significance.

The current version of the portal only displays information about candidate molecules (i.e. genes, miRs, or proteins) from elastic-net analysis. Warning messages will appear if data are not available in the current version. For example, the warning message “this gene was included for elastic-net analysis, but no association with clinical parameters was found” will appear when a user selects a gene that was included in analysis, but not identified as having a relevant association by the elastic-net algorithm. Alternatively, “this gene was not included for elastic-net analysis” indicates that a user has selected a gene that was not included in the analysis. However, the frequency of mutation and CNV by cancers will be provided. Users will view a warning message, “target name not recognized, please try another target name” if they have entered a gene name that does not exist.

**Fig. 4:**
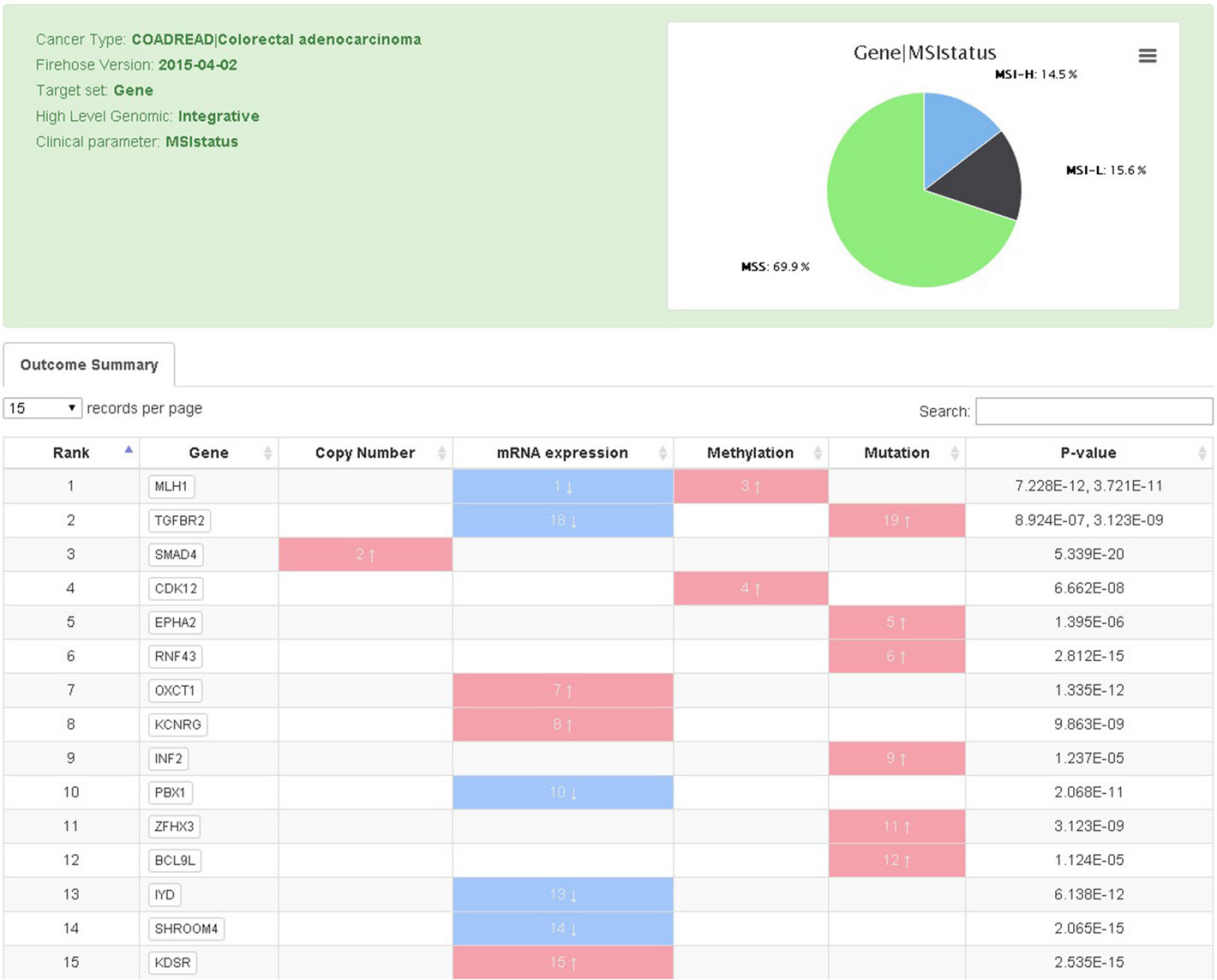
Outcome summary page for clinical stage in COADREAD (colorectal cancer) is reached by selecting a candidate (gene, miR, or protein). The pie chart displays distribution of samples by subtype for the clinical parameter currently selected. This panel shows a table of clinically relevant genes listed by rank and each associated genomic aberration associated with that gene for the clinical parameter and cancer type. For COADREAD and MSI, the gene *MLH1* is top ranked. The color codes of these platforms indicate that *MLH1* often shows decreased gene expression (*downward blue arrow*) and increased methylation (*red upward arrow*)

The second search parameter in the top search panel queries by cancer type (Fig. 2a*ii*). This allows users to select a cancer of interest from a drop-down menu. Once the cancer type is selected, the user can visualize all clinical parameters that are associated with the selected cancer (Fig. 3b; example of COADREAD). In addition, this high-level summary window shows the number of candidates identified by elastic-net analysis for each clinical parameter as well as the total number of samples used for analysis. By clicking on a gene, miR, or protein, users will be directed to an outcome summary page. For example, when a user clicks on “genes” under MSI, the list of genes that are associated with MSI will be displayed in this outcome summary page (Fig. 4). The user has the option to download the relevant information via a download button.

The final search capability in the top search panel permits users to query clinical parameters of interest (Fig. 2a*iii*). Once a clinical parameter is selected from the drop-down menu, a high-level summary page provides a visualization of the number of clinically relevant genes, miRs, or proteins across all cancer types (Fig. 3c; example of clinical stage). From this high-level summary page, the user simply locates the table for the cancer of interest, and then clicks on the gene, miR, or protein. Afterwards, the user is directed to an outcome summary page—it may be noted that this outcome summary page can be reached through different search functions as described earlier.

The outcome summary page offers a variety of useful information (Fig. 4). First, the diagram at the upper right corner shows the distribution of samples by subtype for the clinical parameter currently selected (Fig. 4; example of MSI in colorectal cancer). This diagram, as well as all other figures, can be saved in PNG, JPEG, PDF, or SVG formats by clicking the icon. Second, clinically relevant genes are listed by rank. As previously described, higher-rank genes contribute more to the selected clinical parameter by the supporting genomic platform as derived from elastic-net analysis [25]. In general, genes that are highly ranked for individual category of genetic aberration (e.g. mutations) or across different genomic assays are the most robust and correlate well with other studies, as we noted previously. A blue down arrow—“direct association”—indicates that as the degree of the predictor increases, the outcome increases after controlling for other significant predictors. Likewise, a red upward arrow—“inverse association”—means that as the level of predictor decreases, the outcome increases.

**Fig. 5:**
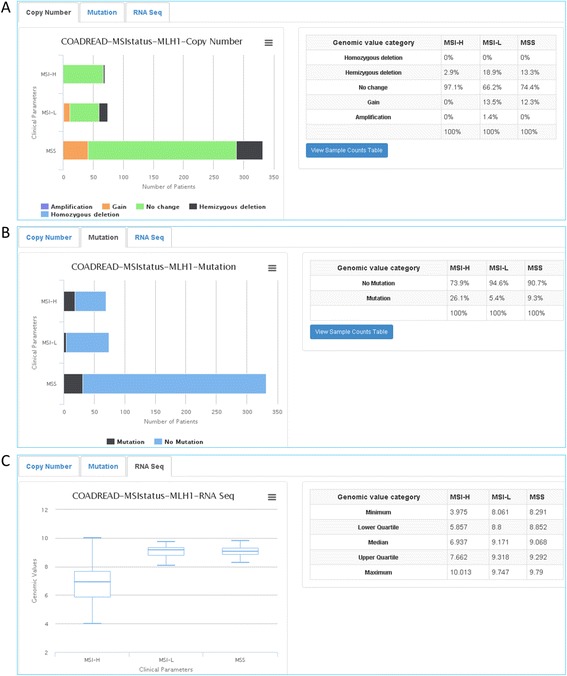
Gene summary page for *MLH1* as a candidate gene associated with MSI. This is reached by selecting a gene, miR, or protein listed in the outcome summary page (Fig. 4). Genomic profile tabs include **a** the status of copy number variation, **b** mutation frequency, and **c** mean expression levels based on RNA-Seq. Genomic profile tabs vary depending on category (gene, miR, or protein), clinical parameter, and cancer type being scrutinized. Tables to the right of the genomic profile graphs display percentiles or quartiles of genomic values for each category in a clinical parameter

Users can also click an individual gene name, which will direct them to a gene summary page (Fig. 5). The gene summary page of *MLH1* displays CNV (Fig. 5a), mutation (Fig. 5b), and mRNA expression levels (Fig. 5c; RNA-Seq displayed, RNA array not displayed). The tabs located above each graph enable users to view different genomic features (copy number, mutation, RNA array, and RNA-Seq) for the gene and parameters selected (Fig. 5a–c).

The companion summary table to the right of the graph displays percentiles for each clinical parameter and genomic category—the sample numbers will only be displayed if the user selects “View Sample Counts Table.” Expression data from RNA-Seq, or RPPA are displayed by box plot and, as a result, summary tables show minimum, first quartile, median, third quartile, and maximum instead of percentiles.

### Profiling a gene, micro RNA, or protein by clinical parameter and cancer type

**Fig. 6:**
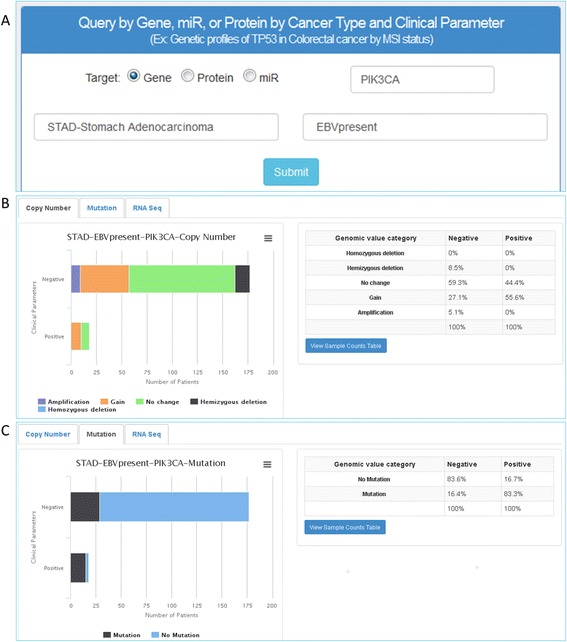
Query and results pages – gene, miR, or protein in a specific cancer type and one clinical parameter. **a** An input query window shows the selections of *PIK3CA* (gene), STAD (cancer type), and EBV present (clinical parameter). Results of genomic profile tabs are shown for (**b**) copy number variation of *PIK3CA* between EBV-infected and EBV-uninfected samples and (**c**) mutation frequency between EBV-infected and EBV-uninfected samples. Genomic profile tabs vary depending on search parameters. Tables to the right of the genomic profile graphs display percentiles for each clinical parameter and genomic value category. This example shows the frequency of *PIK3CA* mutations: 16.4 % of the EBV-negative samples have mutations compared to 83.3 % of EBV-positive samples

The middle search panel allows users to query by gene/miR/protein in a specific cancer with one selected clinical parameter (Fig. 2b). This profiling function requires three inputs including a gene/miR/protein, a cancer type, and a clinical parameter of interest. For example, a user can determine the difference in *PIK3CA* mutation frequency in stomach cancer between patients with EBV infections and patients without EBV infections. To answer this question, users type *PIK3CA* in the gene/miR/protein search box, select STAD for cancer type in the drop-down menu, select EBV presence in the clinical parameter drop-down menu, and click submit (Fig. 6a). A query results page shows the distribution of CNV, the frequency of mutations, and other available genomic/proteomic profiles between EBV-positive and EBV-negative samples (Fig. 6b; copy number, Fig. 6c; mutation). As indicated by the search results, 16.4 % of the EBV-negative samples have mutations on *PIK3CA* while 83.3 % of EBV-positive samples harbor the same mutation (Fig. 6c). Again, the user has the option to use the download button to download a list of relevant genes.

### Test two-hit hypotheses

**Fig. 7:**
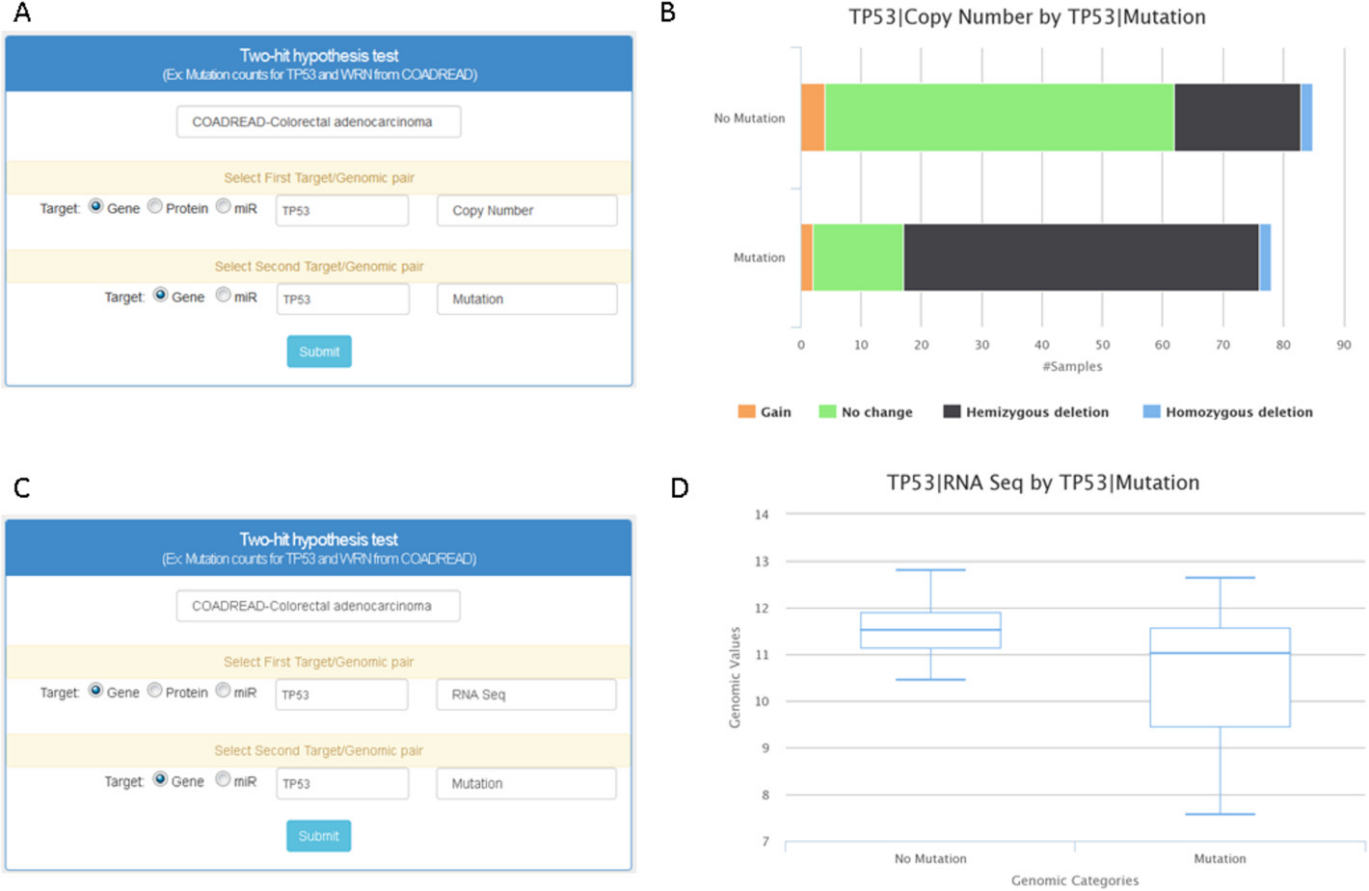
Query and results page – two-hit hypotheses test. **a** The input query window shows the selections of COADREAD (cancer type), *TP53* copy number (first target), and *TP53* mutation (second target). **b** This panel shows the joint copy number status and mutation status for *TP53*—results from the query input are shown in (a). **c** The input query window shows the selections of COADREAD (cancer type), *TP53* RNA-Seq (first target), and *TP53* mutations from genomic sequencing (second target). **d** This panel shows the expression levels of *TP53* in samples with and without mutations as called from the query input shown in (c)

Finally, the bottom search panel called “Two-hit hypothesis test” enables users to explore the relationship between two genomic/proteomic profiles of their choosing (Fig. 2c). This function also allows users to examine how genetic changes affect their corresponding transcriptome/proteome. For example, if a user wishes to know how many samples have *TP53* CNVs overlapping with *TP53* mutations in colorectal cancer, a user selects *TP53* with copy number for the first target and *TP53* with mutation for the second target (Fig. 7a). Once submitted, the query result page provides a graph showing the distribution of CNV of *TP53* between samples with *TP53* mutations and samples without *TP53* mutations (Fig. 7b). This is also summarized in table format (table not displayed). Finally, by selecting RNA-Seq for the first target and mutation for the second target (Fig. 7c; example of *TP53*), the results page will show expression levels by mutation status of the selected candidate gene/miR/protein (Fig. 7d; example of *TP53*). The genomic/proteomic profile for a second target, which splits samples into groups, is limited to mutation and copy number; it is not feasible to split samples by setting an arbitrary cutoff for expression levels. For future updates, we plan to allow users to input their own cutoff to realize the differences above and below cutoffs of their first target sample.

## Conclusions

The Cancer Genome Atlas Clinical Explorer facilitates the clinical use of TCGA data by the broader cancer research and clinical community by providing a simple interface for exploring the clinically relevant associations from TCGA genomic data sets. The search functions provided by this application enhance the clinical utility of TCGA data for biomedical scientist and clinicians. In addition, the Cancer Genome Atlas Clinical Explorer complements existing databases and webpages, such as TCGA data portals, the UCSC Cancer Genomics Browser [23], cBio portal [22], and Broad Firehose, by providing clinically oriented summaries that are easily accessible by a variety of devices including smart phones and laptops.

The TCGA study is ongoing with a significant fraction of samples lacking either genomic results or clinical data. Our elastic-net analysis requires complete data across all of the major genomic assay platforms and clinical annotation; there remain many assay data sets that are incomplete. In addition, some of the cancers are under embargo. We are planning a major upgrade of the portal when the TCGA has final results for all genomics platforms and fully annotated clinical data, and this is likely to occur in 2016. When TCGA results are fully released, we anticipate a benefit from using the completed data sets for a final update. For example, the final release of mutations from the exome data will provide a perfect opportunity to provide comprehensive mutation class and pathogenicity score assignment across all TCGA samples.

## Availability and requirements

Cancer Genome Atlas Clinical Explorer is accessible at http://genomeportal.stanford.edu/pan-tcga. Data can be utilized without any restriction with the citation of this publication.

## Additional files

Additional file 1: Table S1. List of patients with inconsistent clinical outcomes. (XLSX 10 kb) Additional file 2: Table S2. List of cancer genes by COSMIC and TARGET. (XLSX 36 kb) Additional file 3: Table S3. The number of genes selected from TCGA studies by type of genetic alterations and cancer type. (XLSX 9 kb) Additional file 4: Table S4. List of genes/miRs/proteins selected for elastic-net analysis. (XLSX 160 kb) Additional file 5: Table S5. List of genes that cannot be updated properly. (XLSX 8 kb) Additional file 6: Table S6. Conversion of clinical parameters into integer values. (XLSX 9 kb)

## Abbreviations

ACC: adrenocortical carcinoma
BLCA: urothelial bladder cancer
BRCA: breast invasive carcinoma
CESC: cervical cancer
CNV: copy number variation
COADREAD: colorectal adenocarcinoma
COSMIC: Catalogue of Somatic Mutations in Cancer
EBV: Epstein–Barr virus
ERBB2: erb-b2 receptor tyrosine kinase 2
ESCA: esophageal cancer
GBM: glioblastoma multiforme
HER2: human epidermal growth factor receptor 2
HGNC: HUGO Gene Nomenclature
HNSC: head and neck squamous cell carcinoma
JPEG: joint photographic experts group
KICH: chromophobe renal cell carcinoma
KIRC: kidney renal clear cell carcinoma
KIRP: papillary kidney carcinoma
LAML: acute myeloid leukemia
LICH: liver hepatocellular carcinoma
LGG: lower grade glioma
LUAD: lung adenocarcinoma
LUSC: lung squamous cell carcinoma
MLH1: mutL homolog 1
miR: micro RNA
MSI: microsatellite instability
NGS: next-generation sequencing
OV: ovarian serous cystadenocarcinoma
PAAD: pancreatic ductal adenocarcinoma
PCPG: pheochromocytoma and paraganglioma
PDF: portable document format
PIK3CA: phosphatidylinositol-4,5-bisphosphate 3-kinase, catalytic subunit alpha
PNG: portable network graphics
PRAD: prostate adenocarcinoma
RPPA: reverse phase protein array
SKCM: skin cutaneous melanoma
STAD: stomach adenocarcinoma
SVG: scalable vector graphics
TCGA: The Cancer Genome Atlas
TGFBR2: Transforming growth factor, beta receptor II
THCA: thyroid carcinoma
TP53: tumor protein p53
UCEC: uterine corpus endometrioid carcinoma
UCS: uterine carcinosarcoma
WRN: Werner syndrome, RecQ helicase
